# MicroRNA-124-3p Modulates Alpha-Synuclein Expression Levels in a Paraquat-Induced in vivo Model for Parkinson’s Disease

**DOI:** 10.1007/s11064-024-04130-y

**Published:** 2024-03-07

**Authors:** Marta Esteves, Ana Clara Cristóvão, Ana Vale, Marta Machado-Pereira, Raquel Ferreira, Liliana Bernardino

**Affiliations:** 1 CICS-UBI Health Sciences Research Centre, University of Beira Interior, Covilhã, Portugal; 2 NeuroSov, UBImedical, University of Beira Interior, Covilhã, Portugal; 3 Present Address: CEDOC, NOVA Medical School, Faculdade de Ciências Médicas, Universidade NOVA de Lisboa, Lisboa, Portugal; 4 Brain Repair Group, CICS-UBI Health Sciences Research Centre, University of Beira Interior, Av. Infante D. Henrique, Covilhã, 6200-506 Portugal

**Keywords:** miR-124, Alpha-synuclein, Paraquat, Parkinson’s Disease

## Abstract

Parkinson’s disease (PD) is the second most prevalent neurodegenerative disease and the most common movement disorder. Although PD etiology is not fully understood, alpha (α)-synuclein is a key protein involved in PD pathology. MicroRNAs (miRNA), small gene regulatory RNAs that control gene expression, have been identified as biomarkers and potential therapeutic targets for brain diseases, including PD. In particular, miR-124 is downregulated in the plasma and brain samples of PD patients. Recently we showed that the brain delivery of miR-124 counteracts 6-hydroxydopamine-induced motor deficits. However, its role in α-synuclein pathology has never been addressed. Here we used paraquat (PQ)-induced rat PD model to evaluate the role of miR-124-3p in α-synuclein accumulation and dopaminergic neuroprotection. Our results showed that an intranigral administration of miR-124-3p reduced the expression and aggregation of α-synuclein in the *substantia nigra* (SN) of rats exposed to PQ. NADPH oxidases (NOX), responsible for reactive oxygen species generation, have been considered major players in the development of α-synuclein pathology. Accordingly, miR-124-3p decreased protein expression levels of NOX1 and its activator, small GTPase Rac1, in the SN of PQ-lesioned rats. Moreover, miR-124-3p was able to counteract the reduced levels of pituitary homeobox 3 (PITX3), a protein required for the dopaminergic phenotype, induced by PQ in the SN. This is the first study showing that miR-124-3p decreases PQ-induced α-synuclein levels and the associated NOX1/Rac1 signaling pathway, and impacts PITX3 protein levels, supporting the potential of miR-124-3p as a disease-modifying agent for PD and related α-synucleinopathies.

## Introduction

Parkinson’s disease (PD), the most common movement disorder, is characterized by the progressive degeneration of dopaminergic neurons in the *substantia nigra *(SN) of the midbrain, resulting in striatal dopamine depletion. The deficiency of dopamine signaling contributes to both motor and non-motor symptoms [1]. While the etiology of PD is likely multifactorial, the accumulation of cytoplasmic protein inclusions including insoluble α-synuclein (Lewy bodies (LB)) within dopaminergic neurons has been largely recognized as a key pathologic component of the disease. Evidence suggests that misfolded α-synuclein seeds recruit monomeric α-synuclein to form aggregates, which can propagate across brain regions, a phenomenon that correlates with clinical disease progression [2, 3]. These pathologic α-synuclein aggregates are typically hyperphosphorylated at serine 129 [4]. Several factors have been identified to potentiate the formation and propagation of α-synuclein aggregates, including increased expression of α-synuclein, impaired dephosphorylation, decreased clearance, and oxidative stress [5]. However, the precise mechanisms responsible for α-synuclein-induced toxicity and dopaminergic neuronal death are unclear. Evidence suggest that the dysregulation of α-synuclein may increase neuroinflammation, oxidative stress, and mitochondrial dysfunction, leading to dopaminergic degeneration [6]. It is therefore imperative to identify factors that can counteract α-synuclein toxicity. A growing body of evidence has suggested that the dysfunction of miRNA, small non-coding RNA that regulate gene expression at the post-transcriptional level, are promising therapeutic targets for PD [7]. In particular, the levels of brain-enriched miR-124 are decreased in lesioned human brain samples and plasma of PD patients and in PD models [8, 9], suggesting it could be a potential biomarker for the diagnosis of PD. At the molecular level, 24% of miR-124 validated target genes are deregulated in PD [10]. In in vivo PD models, miR-124 overexpression counteracted neurotoxin-induced dopaminergic neuronal injury, oxidative stress, and dysregulated autophagy, while its knockdown had the opposite effect. These effects were associated with the modulation of AMPK/mTOR, p62/p38, annexinA5 (ANXA5), and Bim signaling pathways [11–14]. However, so far, the molecular effects induced by miR-124 are unknown, particularly regarding α-synuclein accumulation and aggregation in an in vivo model of PD.

Herein, we evaluated the role of miR-124-3p in the accumulation of α-synuclein in the paraquat (PQ) rat model of PD. The PQ, a commonly used herbicide, increases α-synuclein levels and aggregation in dopaminergic neurons in the SN, induces degeneration of nigrostriatal dopaminergic neurons, and consequent dopamine depletion in the nigrostriatal pathway, and lipid peroxidation [15, 16]. Our results showed that the intranigral administration of miR-124-3p decreased α-synuclein and phosphorylated α-synuclein at serine 129 (pS129-α-synuclein) protein levels in the SN of PQ-intoxicated rats. Moreover, miR-124-3p counteracted the increased protein levels of NOX1 and its activator Rac1 in the SN triggered by PQ administration. We also showed that PQ reduced protein levels of tyrosine hydroxylase (TH) in the SN and striatum and of pituitary homeobox 3 (PITX3) in the SN. PITX3 is responsible for maintaining the dopaminergic phenotype. Our data showed that miR-124-3p counteracted PQ-induced decrease of PITX3 protein levels in the SN, while no effect was found in TH expression levels. Overall, these results suggest a role for miR-124-3p in regulating α-synuclein levels, likely through modulation of the NOX1/Rac1 signaling pathway, and also regulating the expression of PITX3.

## Materials and Methods

### Animals

Male Wistar rats (3- to 8-month-old) were used in this study. The experimental procedures were performed following protocols approved by the Directorate-General for Food and Veterinary (DGAV), the Portuguese National Authority for Animal Health (21/01/2019; reference number 0421/000/000/2019), and by the Directive 2010/63/EU of the European Parliament and the Council on the protection of animals used for scientific purposes. All animals were maintained in temperature/humidity-controlled conditions under a 12 h light/dark cycle with free access to food and water. All efforts were made to reduce the number of animals used and minimize their suffering.

### Treatment Paradigm

**Fig. 1:**
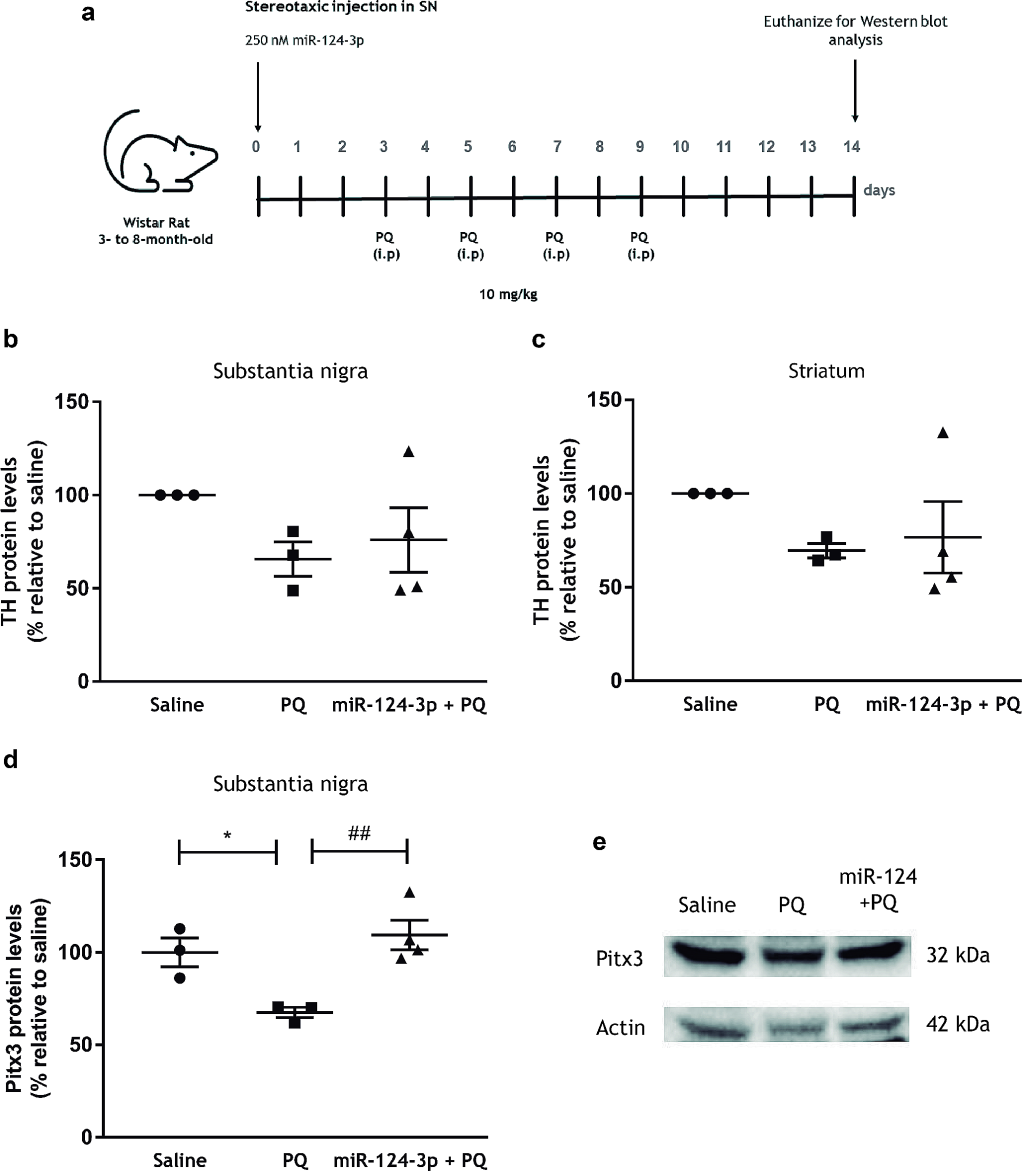
Effects of miR-124-3p in the expression of dopaminergic neuronal markers in the SN and striatum of a rat PQ model in vivo. **(a)** Design and timeline of the experimental animal procedure. Male Wistar rats were subjected to intranigral injection with 250 nM miR-124-3p, or saline, followed by four i.p injections of saline or PQ (10 mg/kg), separated by one day. Five days after the last PQ injection, the brains were collected for western blot analysis. Expression levels of TH in the **(b)** SN and **(c)** striatum, and **(d)**PITX3 in the SN of adult Wistar rats treated with saline, PQ or 250 nM miR-124-3p + PQ. **(e)** Representative western blot protein bands of PITX3 (32 kDa) and actin (42 kDa). TH and PITX3 protein levels were normalized against actin. Data are expressed as a percentage of the controls (saline) ± SEM. Protein expression in the control condition (saline) was set to 100%. *N* = 3 or 4 rats. *p < 0.05 compared to the saline group and ^##^*p* < 0.01 compared to the PQ-treated group using the one-way ANOVA followed by Sidak’s multiple comparison test. Abbreviations: i.p, intraperitoneal; TH, tyrosine hydroxylase; PITX3, pituitary homeobox 3; PQ, paraquat; SN, *substantia nigra*

As depicted in Fig. 1a, each animal received four intraperitoneal (i.p) injections, separated by one day, of either PQ (10 mg/kg of body weight) or sterile saline (0.1 M phosphate-buffered saline (PBS)), according to a protocol described by us [17]. Three days before starting PQ i.p. injections, rats were anesthetized with i.p injection of ketamine (100 mg/kg of body weight) and xylazine (10 mg/kg of body weight) and then stereotaxically injected with 250 nM miR-124-3p (total volume of 2 µL) at the right SN using the following coordinates: mediolateral (ML), − 2.1; anteroposterior (AP), − 5.0; dorsoventral (DV), − 7.7. Three experimental groups were designed: Saline: stereotaxic injection in the SN and i.p injection with saline solution (0.1 M PBS); PQ: stereotaxic injection with saline solution and i.p injection with PQ; miR-124-3p + PQ: stereotaxic injection with miR-124-3p and i.p injection with PQ. Animals were sacrificed five days after the last i.p injection with PQ. For western blot analysis, rats were anesthetized with isoflurane and then sacrificed by decapitation. Brains were collected, and total protein lysates from striatum and the SN were prepared.

### Western Blot

Striatum and SN tissues (ipsilateral side) were lysed on ice in RIPA lysis buffer (50 mM Tris/HCl, pH 8.0, 150 mM NaCl, 5 mM ethylene glycol tetraacetic acid, 1% Triton X-100, 0.5% sodium deoxycholate, 0.1% SDS and 10 mM DDT) with a cocktail of protease and phosphatase inhibitors (Roche Diagnostics Ltd., Mannheim, Germany) by mechanical dissociation. Lysates were then centrifuged, the supernatant collected, and the total protein concentration was quantified using the BCA assay kit (Thermo Scientific) according to the manufacturer’s instructions. Then, 30 µg of total protein was loaded in a 12.5% or 15% polyacrylamide gel, followed by electrophoresis and transfer onto a polyvinylidene difluoride (PVDF) membrane (GE Healthcare, Little Chalfont, UK) for 90 min. Membranes were blocked using 5% milk or 5% bovine serum albumin and then incubated with the following primary antibodies: mouse anti-TH (1:5000; BD Biosciences), rabbit anti-PITX3 (1:1000; Invitrogen), mouse anti-α-synuclein (1:500; BD Biosciences); rabbit anti-pS129-α-synuclein (1:200; Santa Cruz Biotechnology), goat anti-NOX1 (1:200; Santa Cruz Biotechnology), mouse anti-Rac1 (1:750; Millipore), rabbit anti-p47^phox^ (1:1000; Santa Cruz Biotechnology), mouse anti-actin (1:5000; BD Biosciences) and mouse anti-beta-tubulin (1:5000; BD Biosciences); and the secondary antibodies conjugated to horseradish peroxidase—HRP (chicken anti-goat, goat anti-rabbit or goat anti-mouse; 1:5000; all from Santa Cruz Biotechnology). Protein bands were detected by enhanced chemiluminescence (ECL) detection using the ChemiDoc™ MP Imaging System (BioRad Laboratories, CA, USA) and quantified by densitometry analysis using the Image Lab 5.1 software (Bio-Rad Laboratories).

### Statistical Analysis

All data are expressed as mean ± standard error of the mean (SEM) of at least three animals. Statistical analysis was performed using the GraphPad Prism 7.0 Software (GraphPad Software Inc.), using one-way ANOVA followed by Sidak’s or Tukey’s multiple comparison tests. Values of *p* < 0.05 were considered significant.

## Results

### miR-124-3p Impacts Dopaminergic Neurons in the PQ-Induced Model of PD

Recent evidence have shown the neuroprotective role of miR-124 in PD models. Herein, we evaluated the putative neuroprotective effect of miR-124-3p in a PQ-induced PD model. Wistar rats were stereotaxically injected with 250 nM miR-124-3p in the right SN and then exposed to PQ (Fig. 1a). First, dopaminergic neuronal toxicity induced by PQ was investigated by evaluating TH protein levels in the striatum and SN by western blot. We found that PQ exposure reduced TH protein levels in the SN (Fig. 1b) and striatum (Fig. 1c). The administration of 250 nM of miR-124-3p was not able to counteract the reduction of TH levels induced by PQ exposure (Fig. 1b and c). Next, the protein levels of PITX3, which is involved in the survival and maintenance of midbrain dopaminergic neurons, were also evaluated. PQ exposure significantly reduced PITX3 protein levels in SN (Fig. 1d and e; *p* < 0.05) compared to the saline group. The miR-124-3p significantly increased PITX3 protein levels compared to the PQ-intoxicated group reaching levels similar to saline-treated animals (Fig. 1d and e; *p* < 0.01). Altogether, these results show that 250 nM miR-124-3p is not able to counteract PQ-induced decrease of TH protein levels but is able to increase PITX3 protein levels similar to saline animals, suggesting a putative neuroprotective effect.

### miR-124-3p Modulates PQ-Induced α-Synuclein Expression Levels

**Fig. 2:**
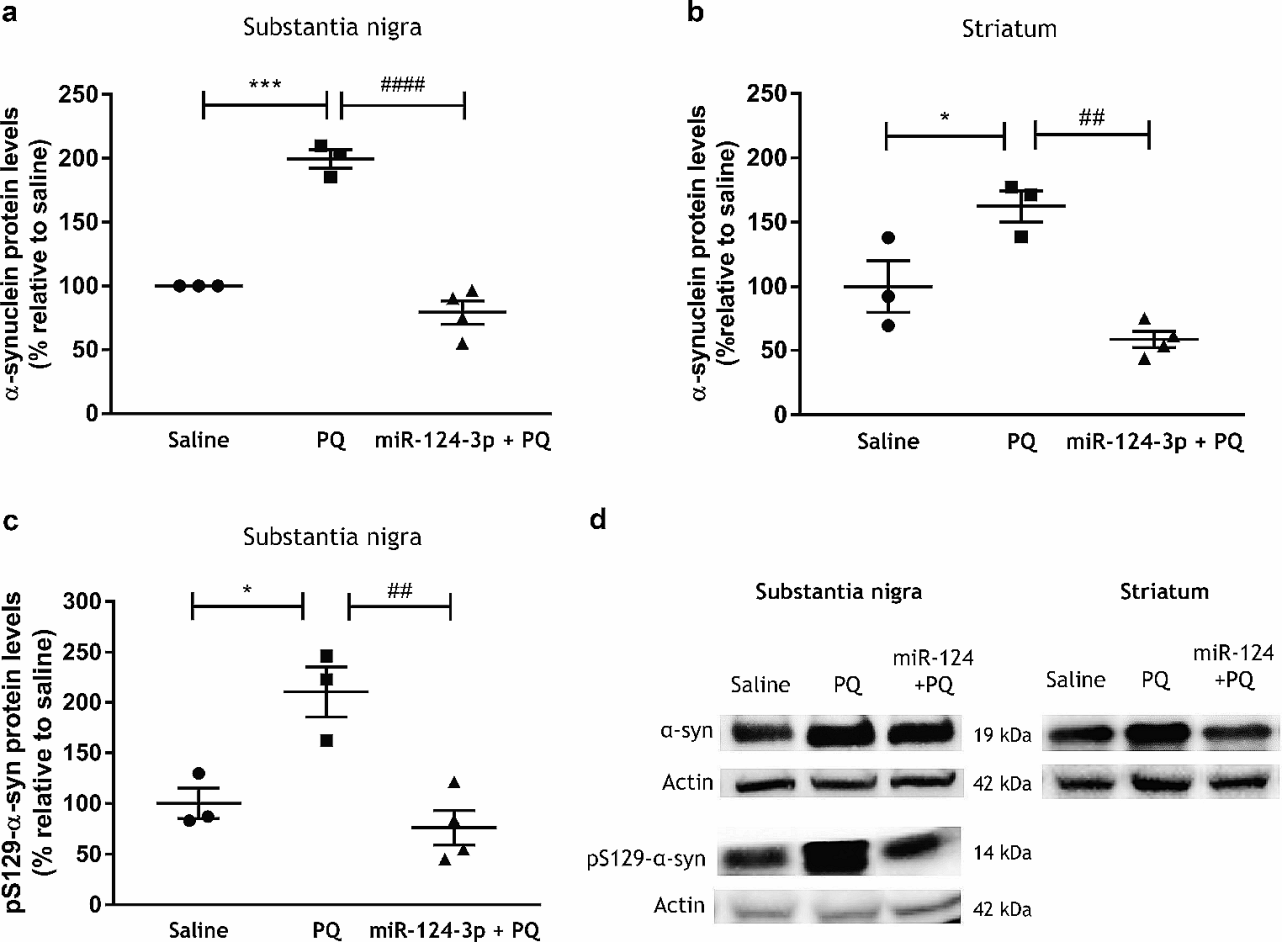
miR-124-3p counteracts the increased levels of total α-synuclein and α-synuclein phosphorylated at serine 129 (pS129-α-synuclein) induced by PQ in vivo. Expression levels of α-synuclein in the **(a)** SN and **(b)** striatum, and **(c)** pS129-α-synuclein in the SN of adult Wistar rats treated with saline, PQ or 250 nM miR-124-3p + PQ. **(d)** Representative western blot protein bands of α-synuclein (19 kDa), pS129-α-synuclein (14 kDa), and actin (42 kDa). α-Synuclein and pS129-α-syn protein levels were normalized against actin. Data are expressed as a percentage of control (saline) ± SEM. Protein expression in the control condition (saline) was set to 100%. *N* = 3 or 4 rats. *p < 0.05 or ***p < 0.001 compared to saline-treated animals and ^##^*p* < 0.01 or ^####^*p* < 0.0001 compared to the PQ-treated group using the one-way ANOVA followed by Sidak’s **(a)** or Tukey’s **(b** and **c)** multiple comparison tests. Abbreviations: α-syn, α-synuclein; pS129-α-syn, α-synuclein phosphorylated at serine 129; PQ, paraquat; SN, *substantia nigra*

To disclose if miR-124-3p affects α-synuclein accumulation, we evaluated the expression of α-synuclein in the ipsilateral SN and striatum of rats exposed to PQ, by western blot. As shown in Fig. 2, PQ exposure significantly increased α-synuclein levels in the SN (Fig. 2a and d; *p* < 0.001) and in the striatum (Fig. 2b and d; *p* < 0.05), when compared with the saline group. The administration of miR-124-3p significantly prevented the increase of α-synuclein levels induced by PQ administration in the SN (Fig. 2a and d; *p* < 0.0001) and striatum (Fig. 2b and d; *p* < 0.01). Next, to investigate if miR-124-3p affects α-synuclein aggregation, we evaluate the expression of pS129-α-synuclein in the SN of rats exposed to PQ. As shown in Fig. 2c, PQ exposure significantly increased the levels of pS129-α-synuclein in the SN (Fig. 2c and d; *p* < 0.05), when compared with the saline group, and the administration of miR-124-3p prevented this increase to levels similar to saline animals (Fig. 2c and d; *p* < 0.01). Therefore, miR-124-3p reduced α-synuclein expression, including the phosphorylated S129-α-synuclein form, induced by PQ in vivo.

### miR-124-3p Counteracts the Increased Levels of NOX1 and Rac1 Induced by PQ

**Fig. 3:**
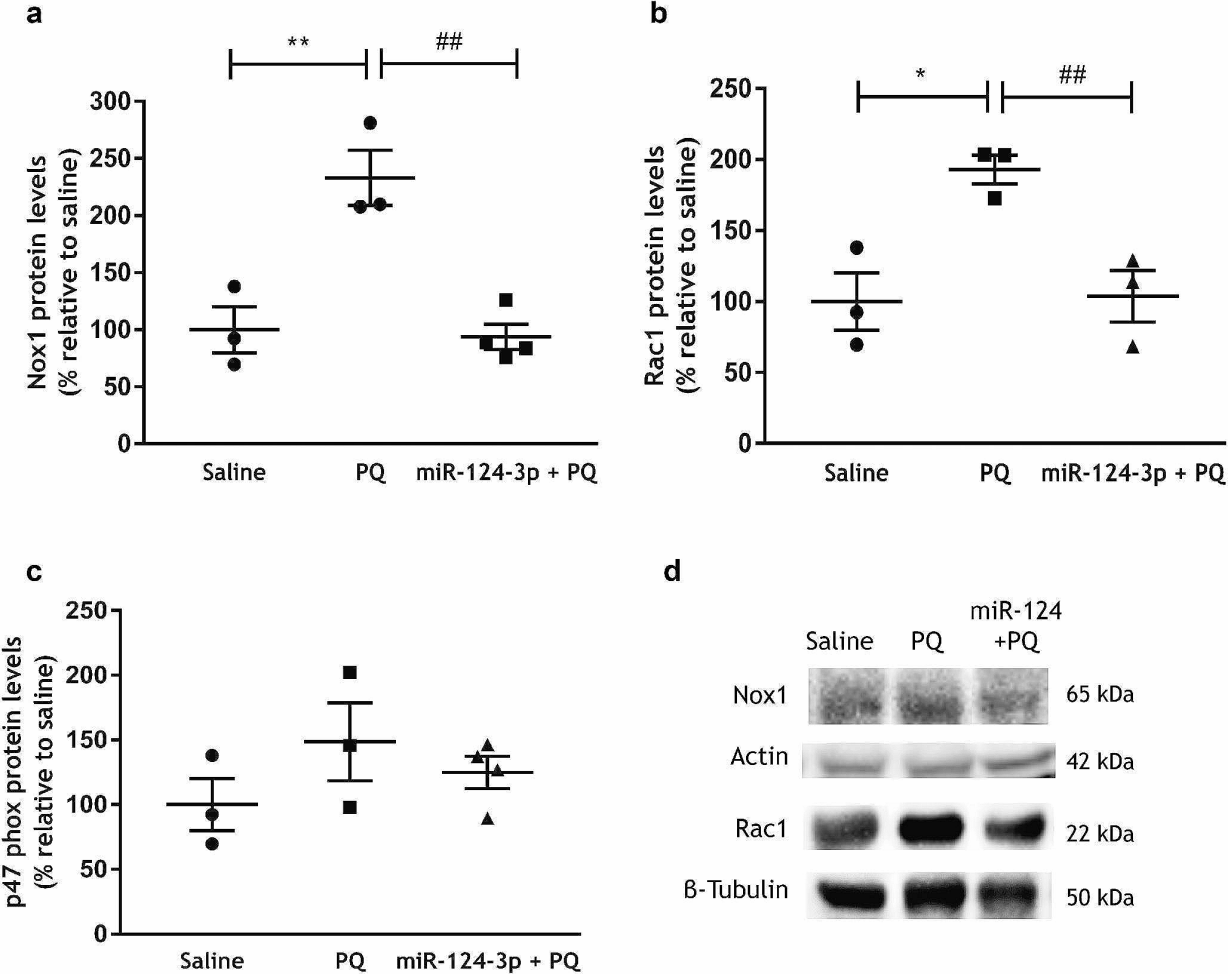
miR-124-3p counteracts the NOX1 signaling pathway induced by PQ in the SN in vivo. Expression levels of **(a)**NOX1, **(b)** Rac1, and **(c)** p47^phox^ in the SN of adult Wistar rat treated with saline, PQ or 250 nM miR-124-3p + PQ. **(d)** Representative western blot protein bands of NOX1 (65 kDa), Rac1 (22 kDa), actin (42 kDa) and β-tubulin (50 kDa). NOX1 and Rac1 protein levels were normalized against actin and β-tubulin, respectively. Data are expressed as a percentage of control (saline) ± SEM. Protein expression in the control condition (saline) was set to 100%. *N* = 3 or 4 rats. *p < 0.05 and **p < 0.01 compared to the saline-treated group and ^##^*p* < 0.01 compared to the PQ-treated group using the one-way ANOVA followed by Sidak’s multiple comparison test. Abbreviations: PQ, paraquat; SN, *substantia nigra*

Nox1-derived oxidative stress has a role in α-synuclein pathology development [15]. Thus, we then disclosed the effect of miR-124-3p in the expression of NOX1, of its organizer Noxo1 homologue, p47^*phox*^, and of Rac1, in the SN of PQ-exposed rats. As expected, administration of PQ induced a significant increase in protein levels of NOX1 (Fig. 3a and d, *p* < 0.01) and Rac1 (Fig. 3b and d, *p* < 0.05) in the SN, compared with the saline-treated group. Contrarily, the intranigral injection of miR-124-3p counteracted the increased protein levels of NOX1 (Fig. 3a and d; *p* < 0.01) and Rac1 (Fig. 3b and d; *p* < 0.01) induced by PQ administration to levels similar to saline-treated rats. Regarding its subunits Noxo1 homologue, p47^*phox*^, neither PQ nor miR-124-3p induced significant alterations in their protein levels compared with the saline-treated group (Fig. 3c). These results suggest that miR-124-3p modulates the NOX1/Rac1 signaling pathway in the PQ-induced PD* in vivo* model.

## Discussion

At the present, there are no effective treatments for the neurodegenerative process in PD. Efforts have been made to establish experimental animal models that recapitulate key pathologic hallmarks to obtain greater insight into the PD pathophysiologic mechanisms and test new therapeutic targets/strategies. The administration of neurotoxins like MPTP, 6-OHDA, rotenone, or PQ or the overexpression of genetically modified genes related to PD, such as SNCA, are the most commonly used models of PD [18]. PQ exposure is considered a key risk factor for sporadic PD in humans and has been widely used to induce in vivo models of PD [17, 19–22]. In this study, we used PQ as a PD model due to its ability to increase α-synuclein levels in the SN [16, 17]. The systemic exposure to PQ in rodents induces oxidative stress, reduction of dopaminergic striatal fibers, and loss of about 36% of dopaminergic neurons in the SN [16, 17, 20]. A large body of evidence highlights α-synuclein dysregulation as the primary cause of various cellular dysfunctions that lead to the degeneration of dopaminergic neurons [2]. In addition, α-synuclein extracted from human LB revealed to be phosphorylated at serine 129 residue. In fact, phosphorylation of α-synuclein at serine 129 was reported to be implicated in the aggregation, LB formation, and consequent dopaminergic neurotoxicity [23]. For this reason, several studies have been trying to identify targets able to counteract α-synuclein pathology and dopaminergic neurodegeneration. So far, no study has analyzed the role of miR-124 in the modulation of α-synuclein in a PD context.

Our study showed for the first time that miR-124-3p is able to down-regulate PQ-induced α-synuclein expression in the striatum and SN. Recent studies have already reported the regulation of α-synuclein levels by other miRNAs. For example, miR-7, which is found decreased in the SN of PD patients, downregulates α-synuclein expression by binding to the *SNCA* mRNA 3’-UTR, thus protecting against neurodegeneration [24]. Therefore, strategies that reduce α-synuclein protein levels might modulate the equilibrium between soluble and misfolded forms leading to gradual clearance of existing pathology. α-Synuclein may contribute to PD pathogenesis in a number of ways, but it is generally thought its oligomeric and fibrillary conformations, the toxic species that mediate disruption of cellular homeostasis, protein clearance pathology and neuronal death [25–28]. While there is no available information about the direct targeting of α-synuclein transcripts, miR-124-3p may target other upstream signaling pathways which are relevant for α-synuclein clearance. For example, miR-124 was reported to alleviate MPTP-induced dopaminergic degeneration by targeting Bim, an apoptosis inducer and autophagy inhibitor [13]. In line, several lines of evidence suggest that autophagy is the major mechanism for the clearance of α-synuclein. Therefore, impaired autophagy leads to increased α-synuclein levels and aggregation which results in dopaminergic degeneration [29]. In particular, PQ induces increased levels of α-synuclein by impairing autophagy function [30, 31]. Therefore, miR-124-3p may reduce α-synuclein levels by boosting autophagy activity. Further studies are needed to understand through which molecular mechanisms miR-124-3p exerts its effect in endogenous and phosphorylated α-synuclein.

NOX, considered one of the main sources of ROS in dopaminergic neurons, has been implicated in the pathology of PD. Specifically, the isoform NOX1, which is upregulated in dopaminergic neurons under oxidative stress has been regarded as a relevant player in dopaminergic neuronal degeneration in PD rodent models [15, 32]. Moreover, NOX1 can be a major player in increasing α-synuclein expression and aggregation and consequent dopaminergic degeneration in both rodent PD models and postmortem human PD brains [15, 17]. The full activation of NOX1 requires the membrane-bound component p22^phox^ and the cytosolic partners Rac1, and NOXo1 and NOXa1 or homologous regulators p47^phox^ and p67^phox^, respectively [33]. Here we showed that the administration of miR-124-3p reduced protein levels of NOX1 and Rac1, a GTPase involved in its activation, in the SN of rats exposed to PQ. The modulation of the NOX1/Rac1 signaling pathway by miR-124-3p may be responsible for the decreased accumulation of α-synuclein in the SN of PQ-exposed rats. In accordance with previous studies [32, 34], we showed that PQ increases the protein levels of NOX1 and Rac1. Although no significant increase in p47^phox^ protein levels was observed, there is an increased tendency in its levels, which is in accordance with the animal model exposed to PQ plus maneb (dithiocarbamate fungicide), which shows an increase in p47^phox^ protein levels [35–37]. Future studies to analyze in depth the effect of miR-124-3p in p47^phox^, p67^phox^ and Rac1 activation will allow to discriminate the modulatory effect on the NOX1 signaling pathway and indirectly in α-synucleinopathy. Additional functional validation studies, such as luciferase reporter and qPCR are needed to disclose if α-synuclein, NOX1 or Rac1 are miR-124 targets. Moreover, while western blot analysis provides total protein expression levels, previously we showed that PQ induced toxicity and oxidative stress in dopaminergic neurons in vitro and in vivo [17, 20]. This acute model induces high systemic toxicity and low survival rates [19, 20]. Hence, future studies aiming to evaluate in depth the effects of miR-124-3p on oxidative stress signaling pathways and its influence on dopaminergic neuronal survival and function should be done using an upgraded PQ exposure paradigm inducing progressive dopaminergic degeneration and consequent motor dysfunction with low systemic toxicity [20].

Previous studies have shown that miR-124 has neuroprotective effects in in vitro and in vivo rodent PD models, attenuating dopaminergic degeneration by targeting several signaling pathways. Therefore, miR-124 attenuated 6-OHDA induced neuronal injury by decreasing apoptosis and reactive oxygen species production through the regulation of the ANXA5/ERK pathway [14]; miR-124 suppression increased neuronal apoptosis and autophagy by regulating AMPK/mTOR signaling pathway in anin vitro MPTP model of PD [12]; overexpression of miR-124 attenuated the expression of the calpain 1/cdk5 pathway proteins improving cell survival in an *in vitro *MPP iodide model of PD [8]; miR-124 upregulated the Hedgehog signaling pathway through inhibition of EDN2 boosting neuronal proliferation and dopamine receptor expression, as well as suppressing neuronal apoptosis in the MPTP mouse model of PD [38]; exogenous delivery of miR-124 agomir inhibited Bim expression, an important protein involved in regulating apoptosis and autophagy process;miR-124 treatment suppressed Bax translocation to mitochondria and lysosome alleviating apoptosis and impaired autophagy in dopaminergic neurons of MPTP mouse model of PD [13]; overexpression of miR-124 in the 6-OHDA-induced PD mouse model ameliorated dopaminergic neuronal loss and improved motor deficits by targeting Axin1 and activating the Wnt/β-catenin pathways [39]. Previously, we also showed that polymeric nanoparticles and small extracellular vesicles loaded with miR-124 were able to trigger neurogenesis and protect dopaminergic neurons, respectively, culminating in the amelioration of motor deficits in the 6-OHDA mouse model of PD [40, 41]. So far, no study has explored the effect of miR-124 in neuronal dopaminergic survival using the PQ-induced PD model, and to strengthen this work, it will be relevant to study the effect of miR-124-3p on the targets described above in this model. In our study, we found that miR-124-3p was able to counteract the reduced protein levels of PITX3 triggered by PQ in the SN. PITX3 is an important transcription factor for dopaminergic neurons specification during neuronal development, being also involved in the maintenance of TH expression and in the survival of dopaminergic neurons throughout adulthood [42]. In addition, it protects dopaminergic neurons against insults by regulating GDNF and BDNF [42, 43]. In PD patients, the number of PITX3-positive neurons is reduced which correlates with dopaminergic neuron loss and accumulation and potential aggregation of α-synuclein. Recently, Wang and colleagues observed an increased accumulation of α-synuclein in the PITX3-deficient dopaminergic neurons of 15-month-old mice, suggesting that the deficiency of PITX3 leads to α-synuclein accumulation which may trigger the pathogenic cascades leading to neurodegeneration [42]. However, our results showed that miR-124-3p is not able to counteract the decrease of TH protein levels induced by PQ, in the striatum and in the SN. Considering the neuroprotective effects of miR-124 reported in other PD models, we may hypothesize that the miRNA concentration and/or the exposure paradigm used in this study are not appropriate to induce an increase of TH expression while is able to increase PITX3 levels similar to controls. Additional quantification of TH-positive neurons in the SN by unbiased stereology and quantification of striatal TH-positive fiber density as well as quantification of dopamine levels in the striatum is also necessary to further confirm its effects in dopaminergic neuronal survival and function. Moreover, the direct delivery of naked miRNA into the brain parenchyma is a limitation for miRNA-based therapies. Due to high susceptibility to degradation by nucleases, and entrapment in the endosome [44], the amount of miRNA reaching targeted regions may not be sufficient to counteract TH reduced levels induced by PQ. In addition, a single administration of miR-124-3p three days before the administration of PQ may also diminish the miRNA effect. However, the mortality rate observed and previously reported by others [20, 45, 46], due to the exacerbated inflammatory response and the systemic toxicity provoked by acute administration of PQ impeded the stereotaxic injection of miR-124-3p during the PQ exposure. However, we have previously shown that the specific biological effect was driven by miR-124-3p, since polymeric nanoparticles loaded with scramble [40] and scramble-enriched small extracellular vesicles [41] have no effects on neurogenesis and neuroprotection in vitro and in vivo. Additionally, the systemic toxicity induced by the acute exposure to the high dose of PQ, lead to non-brain related motor dysfunction, which limits further evaluations of dopamine-dependent motor behaviors. Thus, future experiments using other PD-like synucleinopathy models, depicting no systemic toxicity and high lethality, such as overexpression of human wild-type or mutant α-synuclein using Adeno-Associated Virus (AAV) vectors [47–49] should be used to foster clinical translation of our data. The miR-124-3p was injected in the SN to maximize the amount that reach the lesioned dopaminergic neurons. The delivery of miR-124 by intranasal administration, intravenous or intraperitoneal injections, should be considered in future studies to circumvent the need of an invasive stereotaxic surgery. On the other side, miRNA have short half-life and poor stability which limits its efficient delivery into the brain [50]. To counteract these limitations, we previously developed polymeric nanoparticles and small extracellular vesicles able to efficiently delivery miR-124-3p into the brain, inducing neurogenesis and neuroprotection in a 6-OHDA PD mouse model [41]. Further studies should apply these or other delivery vectors for miR-124-3p in more relevant α-synucleinopathy models. Finally, additional doses of miR-124 should be tested in the future. Despite the need for further studies, here we report for the first time the effect of miR-124-3p on the modulation of α-synuclein expression, the NOX1/Rac1 signaling pathway and PITX3 protein levels in the PQ-induced PD model.

## Conclusions

In conclusion, the present study demonstrates for the first time that miR-124-3p reduces the expression of α-synuclein and downregulates the NOX1/Rac1 signaling pathway in the SN of rats exposed to PQ. These findings suggest that the miR-124-3p is able to counteract α-synuclein-induced pathology, likely through this signaling pathway. Moreover, miR-124-3p was able to counteract PITX3 levels which were decreased in PQ-lesioned rats. Altogether our findings open a new avenue about the role of miR-124-3p as a disease-modifying therapeutic agent for PD and related synucleinopathies.

## Data Availability

The datasets generated during and/or analyzed during the current study are available from the corresponding author upon request.
